# 

**Published:** 1893-06-24

**Authors:** 


PRICE TWOPENCE.

[N]o. 352, Vol. XIV.—June 24th, 1893.

[Registered at the General Post Office as a Newspaper for transmission in the United Kingdom and Abroad.]

WITH EXTRA NURSING SUPPLEMENT.

Per Annum, 8s. 6d., post free.

Monthly Parts, 6d.; post free, 8d.

Ditto per Annum; 8s., post free.

### THE HOSPITAL

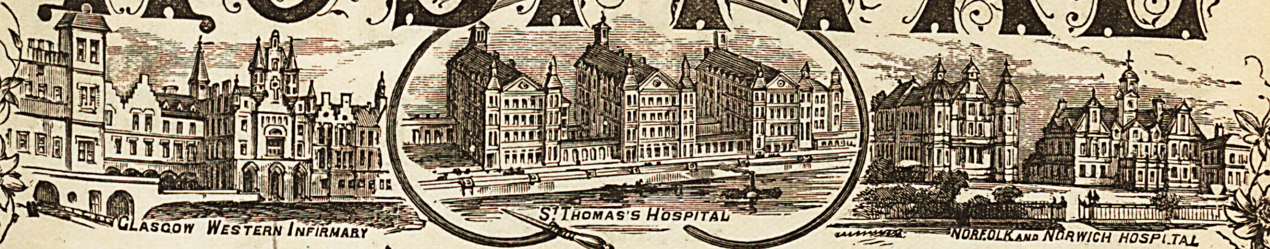

WITH EXTRA NURSING SUPPLEMENT.

No. 352. Vol. XIV.] Saturday, June 24th, 1893. [Twopence.

